# Optimized Epitope-Flanking Sequences Enhance Antigen Presentation and T-Cell Responses Following mRNA Vaccination

**DOI:** 10.3390/cells15191753

**Published:** 2026-09-26

**Authors:** Marie-Pierre Hardy, Gabriel Ouellet-Lavallée, Catherine Thériault, Chantal Durette, Krystel Vincent, Robin Minati, Isabelle Caron, Assya Trofimov, Joel Lanoix, Jean-Philippe Laverdure, Pierre Thibault, Claude Perreault

**Affiliations:** 1 Institute for Research in Immunology and Cancer, Université de Montréal, Montréal, QC H3T 1J4, Canada; marie-pierre.hardy@umontreal.ca (M.-P.H.); gabriel.ouellet.lavallee@umontreal.ca (G.O.-L.); catherine.theriault.4@umontreal.ca (C.T.); chantal.durette@umontreal.ca (C.D.); robin.minati@umontreal.ca (R.M.); i.caron@umontreal.ca (I.C.); joel.lanoix@umontreal.ca (J.L.); jean-philippe.laverdure@umontreal.ca (J.-P.L.); 2 Epitopea, Inc., Montréal, QC H3T 1J4, Canada; krystel.vincent@epitopea.com; 3 Centre de Recherche de l’Hôpital Maisonneuve-Rosemont, Montréal, QC H1T 2M4, Canada; assya.trofimov@umontreal.ca; 4 Cognate Biotechnology Research, Montréal, QC H2S 3H1, Canada; 5 Department of Chemistry, Université de Montréal, Montréal, QC H2V 0B3, Canada; 6 Department of Medicine, Université de Montréal, Montréal, QC H3T 1J4, Canada

**Keywords:** multi-epitope vaccine, mRNA vaccine, tumor-specific antigen, epitope-flanking sequences, MHC-I antigen presentation, immunopeptidomics, CD8^+^ T cells, cancer immunotherapy

## Abstract

mRNA vaccines can encode multiple tumor antigens whose presentation ultimately depends on adequate intracellular processing. To evaluate the potential impact of epitope-flanking sequences, we compared the immunogenicity of two mRNA vaccines in the MC38 mouse model. Both vaccines coded for the same tumor-specific antigens (TSAs). The only difference between the two vaccines was the identity of the epitope-flanking amino acids. The vaccine encoding TSAs bordered by optimized flanking sequences (TSA-Opti) cleared established tumors and elicited robust CD8^+^ T-cell responses. In contrast, the vaccine encoding the same TSAs surrounded by natural flanking sequences (TSA-Nat) induced weaker responses and incomplete protection against tumor growth. Quantitative immunopeptidomics showed that TSA-Opti increased antigen presentation levels, providing a mechanistic link between flanking-sequence design and the enhanced magnitude, clonal diversity, and functionality of TSA-specific CD8+ T cells. TSA-Opti further promoted durable memory, conferring long-term protection against tumor rechallenge. These results identify epitope-flanking sequences as tunable design elements that control peptide–MHC I density and thereby shape the magnitude, clonal diversity, tissue localization, and durability of antitumor CD8^+^ T-cell immunity.

## 1. Introduction

Studies conducted over the last decade have highlighted tumor-specific antigens (TSAs) as promising candidates for cancer vaccines [1,2,3]. TSAs are MHC-associated peptides (MAPs) not found in any normal somatic cells, and they come in two flavors. Mutated TSAs (mTSAs) arise from genomic mutations, whereas aberrantly expressed tumor-specific antigens (aeTSAs) are non-mutant MAPs resulting from cancer-specific epigenetic changes and splicing aberrations [4,5]. Most aeTSAs derive from non-canonical transcripts (introns, UTRs, intergenic regions, etc.) [6,7,8,9]. In contrast to mTSAs, aeTSAs are highly shared among tumors of a given type and may therefore be used to create off-the-shelf vaccines [10,11,12,13,14,15]. The immunogenicity of human aeTSAs has been demonstrated in several models: (i) in vitro priming of PBMCs from normal subjects with aeTSAs elicits the expansion of cytokine-secreting effector CD8 T cells [11,16], (ii) naive CD8 T cells primed against melanoma aeTSAs kill melanoma cells constitutively expressing these aeTSAs [13,17], and (iii) the number of aeTSAs per tumor positively correlates with survival and the abundance of tumor-infiltrating lymphocytes in patients with acute myeloid leukemia [11] and triple-negative breast cancer [12]. Furthermore, in a mouse model (EL4 lymphoma), immunization with dendritic cells (DCs) coated with synthetic aeTSA peptides elicits strong anti-tumor responses [6].

Compared with other cancer vaccine platforms, mRNA vaccines offer notable advantages, including modularity, adjuvanticity, scalability, and safety [1,18,19,20]. Tested mRNA cancer vaccines encode up to 34 discrete peptides that must be adequately processed for presentation as MAPs at the cell surface. This is particularly challenging because multiple intracellular processes can influence antigen processing and presentation. The cytosol contains numerous peptidases that rapidly degrade peptides, with more than 99% of peptides reportedly degraded before reaching the endoplasmic reticulum (ER) [21]. Thus, peptides have a half-life of only 6–10 s in the cytosol of living cells [22]. To accelerate the routing of vaccine-encoded polypeptides into the MHC-I presentation pathway, polyepitope cancer vaccines commonly incorporate an N-terminal leader peptide [23,24,25]. This sequence directs the nascent polypeptide to the ER, providing access to antigen-processing and MHC-I loading pathways in this compartment. However, ER targeting is not required for CD8^+^ T-cell antigen presentation, as cytosolic degradation can also generate proteasome-derived peptides that enter the classical MHC-I pathway. Vaccine-encoded sequences can then contribute to MHC-I presentation through distinct intracellular routes, involving either newly synthesized MHC-I molecules in the ER (the classical pathway) or recycled MHC-I molecules through the vesicular pathway [26]. Then, peptides can be inserted into ER-resident MHC I molecules (the classical pathway) or into recycled MHC I molecules (the vesicular pathway). In the classical pathway, peptides are trimmed by ER peptidases (ACE, ERAP1, ERAP2), then loaded into ER-resident MHC I molecules by a complex comprising TAP1/TAP2 heterodimers, the oxidoreductase PDIA3, and two chaperones (CALR, TAPBP) [27,28,29,30]. For unclear reasons, some endogenous peptides use the alternative “vesicular” pathway at the intersection of ER-derived autophagosomes and early endosomes containing empty (recycling) cell-surface membrane-derived MHC I molecules [29,31,32]. Irrespective of the pathway used, once bound to MHC I molecules, MAPs are protected from peptidases and can be exported to the cell surface.

When CD8 T cells are primed by antigen-presenting cells (APCs), the most crucial limiting factor is MAP density on APCs. Indeed, high MAP abundance enhances CD8 T-cell expansion and functionality [33]. Thus, various strategies are being explored to maximize the abundance of MAPs on APCs following vaccination. In the present study, we investigated whether the immunogenicity of aeTSA-based mRNA vaccines could be modulated by the amino acids flanking the aeTSA epitopes. Evidence suggests that neighboring amino acids can influence the immunogenicity of viral epitopes by modulating proteasomal cleavage [34,35,36,37,38]. However, whether this principle also applies to mammalian proteins remains unclear. In addition, although some amino acid residues are enriched in MAP-flanking sequences, the biological significance of this remains unexplored [39]. Furthermore, any impact of flanking sequences on proteasomal processing would be irrelevant to a vaccine that significantly reduces reliance on cytosolic proteolysis via its signal peptide. Hence, for the time being, MAP-coding sequences in mRNA cancer vaccines are flanked by the amino acids (typically five) naturally found in the MAP source protein.

To directly evaluate the potential impact of epitope-flanking sequences, we compared the immunogenicity of two aeTSA vaccines in the MC38 mouse model: the only difference between the two vaccines was the identity of the epitope-flanking amino acids. In one vaccine, the aeTSA epitopes were flanked by the peptide-specific pentamers found in the source protein; in the second, they were flanked by the pentamers flanking the model ovalbumin (OVA) K^b^-binding SIINFEKL peptide (LEQLE and TEWTS). We selected the flanking sequences of SIINFEKL because this is probably the most extensively studied epitope in T-cell immunology. Indeed, a PubMed search in June 2026 retrieved close to 10,000 articles on “ovalbumin and T cells”. All studies concur that SIINFEKL is processed and presented very efficiently by both the classical and vesicular pathways [40,41,42]. However, we ignore (i) whether LEQLE and TEWTS are instrumental in the efficient processing of SIINFEKL via the classic and/or the vesicular pathway, and (ii) whether these flanking sequences similarly influence the processing of heterologous peptides. The present study shows that OVA flanking sequences enhance the presentation of MC38 aeTSAs. Following vaccination, OVA flanking sequences increased the number, functionality, and repertoire diversity of aeTSA-responsive CD8 T cells. We refer to this construct as TSA-Opti, as OVA-derived flanking sequences were associated with improved presentation and immunogenicity relative to the natural flanks in the present system.

## 2. Materials and Methods

### 2.1. Cell Lines and Animals

The MC38 colorectal cancer cell line, specifically the diploid MC38-K line [43], was obtained from Kerafast (Boston, MA, USA). MC38 cells were cultured in DMEM supplemented with 10% FBS, 10 mM HEPES, 1× nonessential amino acids, and 1% penicillin-streptomycin. The DC2.4 dendritic cell line was purchased from Sigma-Aldrich (Saint Louis, MO, USA) and was cultured in RPMI-1640 supplemented with 10% FBS, 10 mM HEPES, 1× L-Glutamine, 1× nonessential amino acids, 1% penicillin-streptomycin, and 50 μM β-mercaptoethanol.

C57BL6/J mice (strain #000664, 8–12 weeks, 20–25 g) were purchased from Jackson Laboratory (Bar Harbor, ME, USA). Mice were group-housed (2–4 per cage) and maintained in ventilated cages under standard conditions (23 ± 1 °C, 55 ± 5% humidity, 12 h light/dark cycle) in the in vivo specific pathogen-free facility at IRIC (University of Montreal). Mice had access to water and food ad libitum and were allowed to acclimatize for at least 7 days before the start of experimental procedures. All animal procedures were performed in accordance with the animal welfare committee of the University of Montreal (Comité de Déontologie en Expérimentation Animale, CDEA) and the regulations of the Canadian Council on Animal Care (CCAC).

### 2.2. RNA Extraction, Library Preparation, and Sequencing

One replicate of 2 × 10^6^ MC38 cells was used for RNA-Seq. RNA isolation via TRIzol, purification, and quantitation of high-quality RNA samples were performed as previously described [6]. The MC38 cDNA library was prepared from isolated RNA and amplified by PCR before sequencing. Paired-end RNA-seq was performed on an Illumina (San Diego, CA, USA) NextSeq500 and yielded 254 × 10^6^ reads.

### 2.3. Database Generation for Shotgun Mass Spectrometry

#### 2.3.1. Generation of the Canonical Proteome

This was conducted as previously described [6]. Briefly, RNA-seq reads were trimmed to remove sequencing adapters and low-quality 3′ bases using Trimmomatic version 0.35, then aligned to the reference genome (GRCm38.99) using STAR version 2.5.1b. Transcript expression was quantified in transcripts per million (tpm). To build the MC38 canonical proteome, pyGeno was used to insert high-quality, sample-specific single-base mutations into the reference exome and to export sample-specific sequence(s) of known proteins derived from expressed transcripts (tpm > 0). Following in silico translation of these sequences in their annotated reading frame, the resulting protein sequences were written to a fasta file.

#### 2.3.2. Generation and Filtering of K-Mer Database and Cancer-Specific Proteome

This was performed as previously described [6], with the following exceptions: 8 mTEC samples (GEO accessions GSE127825, GSE127826) were used instead of 6, and the k-mer occurrence allowed in mTECs was 1 instead of 0. Briefly, R1 and R2 fastq files were trimmed as reported above, and the reverse mapping reads (R2) were reverse complemented using the fastx_reverse_complement function of the FASTX-Toolkit v0.0.14. K-mer databases (24- or 33-long) were generated using Jellyfish v2.2.3 [44]. Extraction of 33-nucleotide-long k-mers that could give rise to tumor antigens (TAs) was performed as previously described, including the application of a threshold (observation at least 4× in MC38) to exclude sequencing errors and excluding k-mers with occurrence >1 in the mTEC k-mer database. The remaining k-mers were assembled into contigs using NEKTAR, an in-house-developed software. Finally, we 3-frame-translated the contigs using an in-house Python 3 script, split amino acid sequences at internal stop codons, and concatenated the resulting subsequences with the canonical proteome. Canonical and cancer-specific proteomes were then concatenated and subsequently used for mass spectrometry (MS) database search and peptide classification.

### 2.4. Isolation of MC38 MHC I Peptides and Shotgun Mass Spectrometry

One replicate of 240 × 10^6^ cells was prepared from exponentially growing cells. Samples were lysed in a buffer containing 1% *w*/*v* CHAPS, and MHC I peptides were immunoprecipitated, as previously described [45], using covalently cross-linked MHC Class I Pan H-2 (M1/42.3.9.8), H-2Db (24-14-8S), and H-2Kb (Y3) antibodies (BioXcell, Lebanon, NH, USA) to CNBr Sepharose beads. Dried peptide extracts were re-suspended in 0.2% formic acid and separated on C18 Jupiter Phenomenex. Samples were analyzed with an Exploris 480 (Thermo Fisher Scientific, San Jose, CA, USA) instrument in positive-ion mode using a Nanoflex source at 2.8 kV. Each full MS spectrum, acquired with a resolution of 240,000, was followed by 20 MS/MS spectra, where the most abundant multiply charged ions were selected for MS/MS sequencing with a resolution of 45,000, an automatic gain control target of 100%, an injection time of 1000 ms, and a collisional energy of 34%. Peptides were identified using Peaks X 10.6 (Bioinformatics Solution Inc., Waterloo, ON, Canada), and peptide sequences were searched against the MC38 global cancer database using a peptide-identification tolerance of 10 ppm and a precursor-ion tolerance of 0.01 Da. Oxidation (M) and deamidation (NQ) were considered as post-translational modifications.

### 2.5. Identification of MHC I-Associated Peptides and Selection of Tumor Antigens

Peptide-spectrum matches (PSMs) from Peaks were filtered at a 5% false discovery rate (FDR). Resulting unique peptides were restricted to 8–11 amino acids and required to have a predicted eluted ligand percentile rank (%Rank_EL) ≤ 2 for at least one relevant MHC I molecule, as determined by NetMHCpan 4.1b [46].

TA candidates were selected based on their source RNA expression in the cancer sample of origin and mTECs. MAPs were retained as TA candidates if all possible MAP-coding sequences for a given MAP (i) were expressed below 2 KPHM (minimum occurrence of the MAP-coding sequence’s 24-nucleotide-long k-mer set per hundred million reads) in mTECs and (ii) had a KPHM fold change greater than or equal to 10 in cancer compared to mTECs. Because leucine (L) and isoleucine (I) variants are not distinguishable by standard MS approaches, TA candidates for which an existing I/L variant was flagged as a non-TA candidate were discarded unless the TA had higher RNA expression than the I/L variant in the tumor sample. Finally, genomic locations for all MS-validated TA candidates were identified by mapping reads containing MHC peptide-coding sequences to the reference genome (GRCm38.99) using BLAT (UCSC Genome Browser). TA candidates for which reads did not match to a concordant genomic location or matched to hypervariable regions (such as the MHC, Ig, or TCR genes) were excluded.

As described previously [6], the peripheral expression of TA candidates’ peptide-coding sequences was assessed using RNA-seq data from 22 murine tissues sequenced by the ENCODE consortium [47]. aeTSA candidates meeting both of the following criteria were considered as genuine aeTSAs:

1. Expression in MC38 ≥ 2 × Encode+mTECs 90th Percentile Value
2. Mean Expression in mTECs and Other Peripheral Tissues (Except Testis) ≤ 2.5 RPHM

Antigen-encoding sequences meeting the first criterion but expressed in mTECs or other peripheral tissues > 15 RPHM were designated as TAAs.

### 2.6. MS Validation of TSA Candidates

Following MAP identification with PEAKS, TA candidates were further validated using these methods:

(1) LC-MS/MS data were searched against the relevant database using Comet 2022.01 rev 0 [48], using the same parameters used in PEAKS. A 5% FDR as determined by Percolator v3.4 [49] (features: mass, mass error, charge, sequence length, ions matched and total, Comet e-value/Xcorr/deltaCN/sp score) was applied to all peptide-spectrum matches.
(2) MS spectra of all TA candidates that were found in the Comet database search were submitted to an in-house manual validation to remove any false identification.

Thus, all TAs listed herein were re-identified using a second MS search algorithm (Comet) and subsequently validated manually.

### 2.7. Vaccine Design

To design a proof-of-concept construct for our improved mRNA vaccine strategy, we selected five of the identified MC38 aeTSAs. These five antigens were arranged in a string-of-beads manner. Each antigen “unit” consisted of the minimal MHC I epitope bordered by five amino acid flanking regions on both sides. These units were linked together by a flexible chain of amino acids. We built two construct variants that differed only in the amino acids flanking the aeTSAs (Figure 1A).

In the first construct, the aeTSAs were bordered by their naturally occurring flanking sequences from their predicted source transcript. In the second construct, we used the flanking sequences of the well-studied SIINFEKL peptide to substitute for the natural flanking sequences of the MC38 epitopes. The five amino acids flanking the SIINFEKL peptide at both the N- and C-termini (LEQLE and TEWTS, respectively) in its source protein were obtained using the reference sequence available from the National Library of Medicine (Gene ID: 396058, Protein accession number: NP_990483.2). A third construction using five TAAs flanked by their naturally occurring flanking sequences was also designed.

The open reading frame encoded two other elements in addition to tumor antigens. The first is the signal peptide of the protein calreticulin, which directs the nascent polypeptide to the ER. The second is PADRE, an MHC II epitope that engages CD4+ T cells, thereby providing CD4+ T-cell help to the immune response [50].

Our designed open reading frames were codon-optimized by eTheRNA (Niel, Belgium), which also added proprietary 5′ and 3′ UTRs. The nucleotide sequences of the vaccine ORFs are provided in Table S1. No nucleoside modifications were incorporated during mRNA manufacture. The lipid nanoparticles (LNPs) contained a proprietary lipid mixture that included an ionizable lipid from NOF Corporation (Tokyo, Japan). These LNPs are efficiently delivered to the spleen (rather than the liver) due to their diameter (approx. 120 nm) [51]. LNP formulations were stored in 2 mL sterile glass vials containing 1 mL of LNP formulation at approximately 100 µg/m. Vials were kept at −80 °C.

### 2.8. Vaccination Protocol and Tumor Growth Studies

MC38 cells (1 × 10^5^) in a 100 μL volume were administered by subcutaneous injection under light anesthesia (1.5% oxygen and 2% isoflurane). Mice (*n* = 10 per group) received 3 intravenous doses of mRNA-LNPs through their lateral tail vein at one-week intervals, starting either 4 days after (therapeutic experiment) or 21 days prior to (prophylactic experiment) MC38 inoculation. More specifically, 10 µg of LNP-mRNA were diluted to an exact concentration of 50 µg/mL using a 20 mM Tris, 0.9% NaCl dilution buffer. A 200 µL injection volume was used for administration.

Tumor size was measured twice per week using a standard manual caliper, starting 7 days following cell graft. Tumor volume (mm^3^) was estimated using the formula: volume = (length × width^2^)/2. Mice presenting with a tumor diameter exceeding 10 mm, a loss of weight greater than 20%, tumor ulcerations or necrosis, or tumors interfering with the mouse’s normal function were euthanized by CO_2_ inhalation.

### 2.9. Evaluation of CD8+ T-Cell Responses Induced by mRNA-LNPs

#### 2.9.1. Vaccination and Cell Preparation

Mice received three doses of the LNP-mRNA vaccines administered intravenously at one-week intervals, as described above. A subset of mice received a booster dose two months after the third vaccination. Unless otherwise specified, only spleens were harvested following the last vaccination. In some experiments, pooled spleens and lymph nodes were used. Tissues were mechanically dissociated using syringe plungers and 70 µm mesh filters. Bone marrow from the femurs and tibias of mice at 60 days post third vaccination was flushed with a syringe fitted with a 26 g needle and RPMI media (Cat. 11875-093, Gibco, Carlsbad, CA, USA). Red blood cells in splenocyte and bone marrow suspensions were lysed with RBC lysing solution (Cat. 420331, BioLegend, San Diego, CA, USA) according to the manufacturer’s instructions. RBC-depleted cell suspensions from the spleen, lymph nodes, and bone marrow were then either used directly for T-cell phenotype staining or subjected to CD8+ T-cell enrichment using the Mouse CD8^+^ T-cell Isolation Kit (cat. 130-104-075, Miltenyi Biotec, Gaithersburg, MD, USA) according to the manufacturer’s instructions. For all FACS experiments, cells were stained for viability assessment with Zombie Aqua Fixable viability dye (Cat. 77143, BioLegend) for 15 min at room temperature, and nonspecific antibody binding was prevented using Mouse BD FC Block (Cat. 553141, BD Biosciences, Mississauga, ON, Canada). Samples were analyzed on a FACSFortessa™ flow cytometer (BD Biosciences).

#### 2.9.2. T-Cell Phenotyping

Splenocytes obtained as previously described were stained with the following antibodies for 30 min at 4 °C: BV421 anti-CD3 (BD Horizon, Cat. 562600), APC-Cy7 anti-CD4 (BD Pharmingen, Cat. 552051), BV711 anti-CD8 (BD Horizon, Cat.563046), FITC anti-CD44 (BD Pharmingen, Cat. 553133), AlexaFluor 700 anti-CD62L (BD Biosciences, Cat. 560517), APC anti-CD25 (BD Pharmingen, Cat. 557192). Intracellular staining was performed with FoxP3 buffer set (Cat. 00-5523-00, eBioscience Inc., San Diego, CA, USA) and PE anti-FoxP3 (Cat. 130-111-600, Miltenyi Biotec).

#### 2.9.3. CD8^+^ Dextramer Staining

To assess their expansion and phenotype, magnetically enriched CD8+ T cells were stained for TA specificity using U-Load dextramers folded according to the manufacturer’s instructions (Cat. U-LX41 for H-2 Db-binding TAs, U-LX42 for H-2 Kb-binding TAs, Immudex, Copenhagen, Denmark). For each TA specificity, dextramers were loaded with PE and APC fluorochromes separately. One million CD8^+^ T cells enriched from the spleen or two million total bone marrow cells were stained with both PE- and APC-loaded dextramers simultaneously for 10 min at room temperature. Only double-positive CD8+ T cells were considered TA-specific. Cells were subsequently stained with the following antibodies for 30 min at 4 °C: anti-CD8, anti-CD44, anti-CD62L as described above, as well as BV711 anti-PD1 (BD Horizon, Cat. 568298) and BV421 anti-LAG3 (BD Optibuild, Cat. 740072). Samples were analyzed on a FACSFortessa™ flow cytometer (BD Biosciences).

#### 2.9.4. IFN-γ ELISpot Assays

To assess the functionality of CD8+ T cells induced by the LNP-mRNA vaccines, we performed IFN-γ ELISpot assays using an R&D Systems kit (cat. EL485, R&D Systems, Minneapolis, MN, USA), as previously described [6]. Sorted CD8+ T cells from the spleen of vaccinated mice were plated and incubated at 37 °C for 42–44 h in the presence of irradiated splenocytes (4000 cGy) from syngeneic naïve mice pulsed with the relevant peptide (4 μM). As negative controls, CD8+ T cells from mice vaccinated with empty LNPs were incubated with peptide-pulsed splenocytes. The plates were incubated for 40–44 h at 37 °C, then washed and developed according to the manufacturer’s protocol. Spots were counted using an AID Classic ELISpot Reader (AID, Strassberg, Germany). IFN-γ production was expressed as the number of spot-forming units per 10^6^ CD8+ T cells.

### 2.10. DC2.4 Lipofection and Targeted MS Analyses

To evaluate the impact of flanking sequences on antigen presentation, we established an in vitro assay using DC2.4 cells, a murine dendritic cell line. As an initial step, DC2.4 cells were pulsed with 2 μg/mL of each synthetic TSA analog to assess peptide binding capacity, while untreated cells served as a negative control. MHC I-associated peptides were isolated and analyzed by targeted mass spectrometry to quantify baseline presentation.

DC2.4 cells were then lipofected with the different mRNA-LNP constructs (1.25 μg per 10^6^ cells). Briefly, on the day of the experiment, DC2.4 cells were washed once with 1× PBS and detached using TrypLE™ Express Enzyme (cat. 12605-028, Gibco). After counting, cells were seeded at a density of 1.4–1.7 × 10^5^ cells/cm^2^ in 30 mL of culture medium in T175 flasks. Cells were allowed to reattach for at least 3 h prior to lipofection with mRNA-LNPs. The required mRNA-LNP volume was diluted in 2 mL of culture medium and added to the culture flask. Cells were harvested 6 and 18 h post-lipofection after washing once with 1× PBS, and MHC-I-associated peptides were isolated by immunoprecipitation (see Section 2.4).

TSA copy number per cell was quantified by targeted mass spectrometry using ^13^C-labeled synthetic peptide analogs as internal standards, together with an external calibration curve generated from increasing concentrations of unlabeled peptides. Endogenous TSA signals were accepted only when co-eluting fragment-ion transitions matched those of the corresponding heavy-isotope-labeled standards. Copy numbers were derived from the calibration curve and normalized to the number of cells processed for MHC I immunoprecipitation. For each peptide, the lower limit of detection and lower limit of quantification were defined from the calibration curve and used to distinguish true absence from signals below the quantitative range.

To assess whether flanking sequence optimization generated alternative MHC class I ligands, the complete TSA-Opti and TSA-Nat coding sequences were screened in silico with NetMHCpan 4.1b to predict 8–11-mer binders to H2-Kb and H2-Db. Predicted binders (%Rank_EL ≤ 2, *n* = 27) were compiled into a targeted LC–MS/MS inclusion list (Table S2), which was used to interrogate MHC I peptide datasets for potential alternative vaccine-derived ligands.

### 2.11. Single-Cell RNA Sequencing and V(D)J Profiling

#### 2.11.1. Preparation of Samples and Sequencing

Single-cell RNA sequencing (scRNA-seq) coupled with V(D)J profiling was performed to characterize the transcriptional and clonal landscape of antigen-specific CD8^+^ T cells. Mice received three intravenous doses of TSA-Nat (*n* = 11) or TSA-Opti (*n* = 3) RNA-LNP vaccines at one-week intervals. Spleens were harvested seven days after the final immunization and processed as described in Section 2.9.1. Dextramer-labeled CD8^+^ T cells specific for TSA2 and TSA4 were sorted on a BD FACSAria cell sorter (BD Biosciences) at >95% purity and high sheath pressure. Isolated cells were subjected to single-cell library preparation using the 10x Genomics Chromium Single Cell 5′ Gene Expression and V(D)J platform (10x Genomics, Pleasanton, CA, USA) according to the manufacturer’s instructions, enabling simultaneous transcriptome and T-cell receptor (TCR) profiling. Libraries were sequenced on a NextSeq 2000 platform (Illumina, San Diego, CA, USA) using paired-end 100 bp reads.

Gene expression libraries contained ~500 million reads per sample (median across samples), with ~80% of reads confidently mapping to mouse transcripts. Of these transcript-mapped reads, ~80% aligned to exonic regions, consistent with high-quality single-cell RNA capture. V(D)J libraries contained ~60 million reads per sample, with a mean of ~25,000 reads per cell (10,000–15,000 reads per cell retained after filtering for productive TCR sequences).

#### 2.11.2. Data Processing and Analyses

Raw sequencing reads were processed using Cell Ranger (v8.0.1, 10x Genomics, Pleasanton, CA, USA) with the multi pipeline for joint analysis of 5′ gene expression and V(D)J libraries, including alignment to the mouse reference genome, gene quantification, and TCR sequence assembly. Downstream analyses were conducted in R using Seurat [52]. Low-quality cells were excluded based on the following criteria: nFeature_RNA < 500 or >5000, nCount_RNA > 25,000, or mitochondrial transcript content > 5%.

After preprocessing and filtering, gene expression data were normalized and scaled, then subjected to principal component analysis (PCA) for dimensionality reduction. The top 10 principal components were used for graph-based clustering using the Louvain algorithm in Seurat. Clusters were identified at a resolution of 0.5, and Uniform Manifold Approximation and Projection (UMAP) [53] was used for two-dimensional visualization of the cellular landscape. Cluster 9 (*n* = 23 cells), exhibiting high mitochondrial content and low gene counts, indicative of low-quality cells, was excluded from downstream analyses. Portions of the R scripts used for single-cell RNA-sequencing analysis were developed with the assistance of an AI-based tool (ChatGPT, GPT 5.2 Instant, OpenAI). The code was validated and adapted by the authors.

#### 2.11.3. Opti vs. Nat Differential Gene Expression Analysis

For differential expression analysis, pseudobulk profiles were generated by aggregating gene counts across all cells within each biological condition. Genes with total counts ≤ 10 across all 4 samples were excluded. Differential gene expression between TSA-Opti and TSA-Nat conditions was assessed using DESeq2 [54], modeling epitope as a covariate to estimate a shared vaccine effect across epitopes. Genes with an absolute log_2_ fold change > 0.5 and an adjusted *p*-value < 0.05 were considered significant. For heatmap generation, a curated panel of genes related to CD8^+^ T-cell differentiation and function was combined with differentially expressed genes identified from the DESeq2 pseudobulk analysis. Expression values were averaged per sample and Z-scored across genes to highlight relative transcriptional differences between conditions.

#### 2.11.4. TCR Diversity

TCR β-chains (TRB), more specifically CDR3β sequences, were analyzed to assess clonal diversity and expansion within antigen-specific CD8^+^ T cells. For these analyses, only cells with a single productive TRB chain were retained, yielding between 1780 (Nat_S4) and 3527 (Opti_S4) cells per condition. Clonal diversity was quantified using Shannon entropy and inverse Simpson indices, and the D50 metric was calculated to determine the number of dominant clonotypes that together account for 50% of the repertoire. Prior to diversity estimation, clonotype repertoires were normalized by random subsampling to an equal number of cells per condition (*n* = 1000 cells per subsample), and this procedure was repeated across bootstrap iterations. Statistical significance of differences between conditions was assessed using bootstrap resampling of the normalized clonotype distributions.

### 2.12. Isolation of Tumor-Infiltrating Lymphocytes (TILs)

MC38 inoculation was performed as described in Section 2.8. Mice were vaccinated on days 7, 14, and 21 post-inoculation with one of the two TSA vaccines. Mice were euthanized, and tumors were collected 22 days post-inoculation. Any fat and necrotic regions were removed from the tumors. The tumors were then dissociated using the mouse tumor dissociation kit (Cat. 130-096-730, Miltenyi Biotec) according to the manufacturer’s instructions and the “37c_m_TDK_1” program on the gentleMACS™ Octo Dissociator with Heaters (Miltenyi Biotec). CD45+ cells were magnetically enriched from the tumor cell suspension by using the mouse CD45 (TIL) microbeads (Cat. 130-110-618, Miltenyi Biotec) according to the manufacturer’s instructions. CD45+ cells were then subjected to FACS analysis as described in Section 2.9.2 and Section 2.9.3, using antigen-specific dextramers and antibodies against CD3, CD4, CD8, CD44, and CD62L.

## 3. Results

### 3.1. Identification of Tumor Antigens and mRNA Vaccine Design

Using the MHC I TA identification workflow described in the methods, we identified five aeTSAs and five TAAs in the MC38 cell line (Table 1). To test whether the immunogenicity of aeTSA-encoding mRNA vaccines could be modulated by epitope-flanking sequences, we compared two designs (Opti and Nat) that differed only in the amino acids flanking the epitopes (Figure 1A). mRNA constructs encoding the five aeTSAs were synthesized using both designs (TSA-Opti and TSA-Nat). An additional construct encoding the five TAAs with the Nat design (TAA-Nat) was also generated.

**Figure 1:**
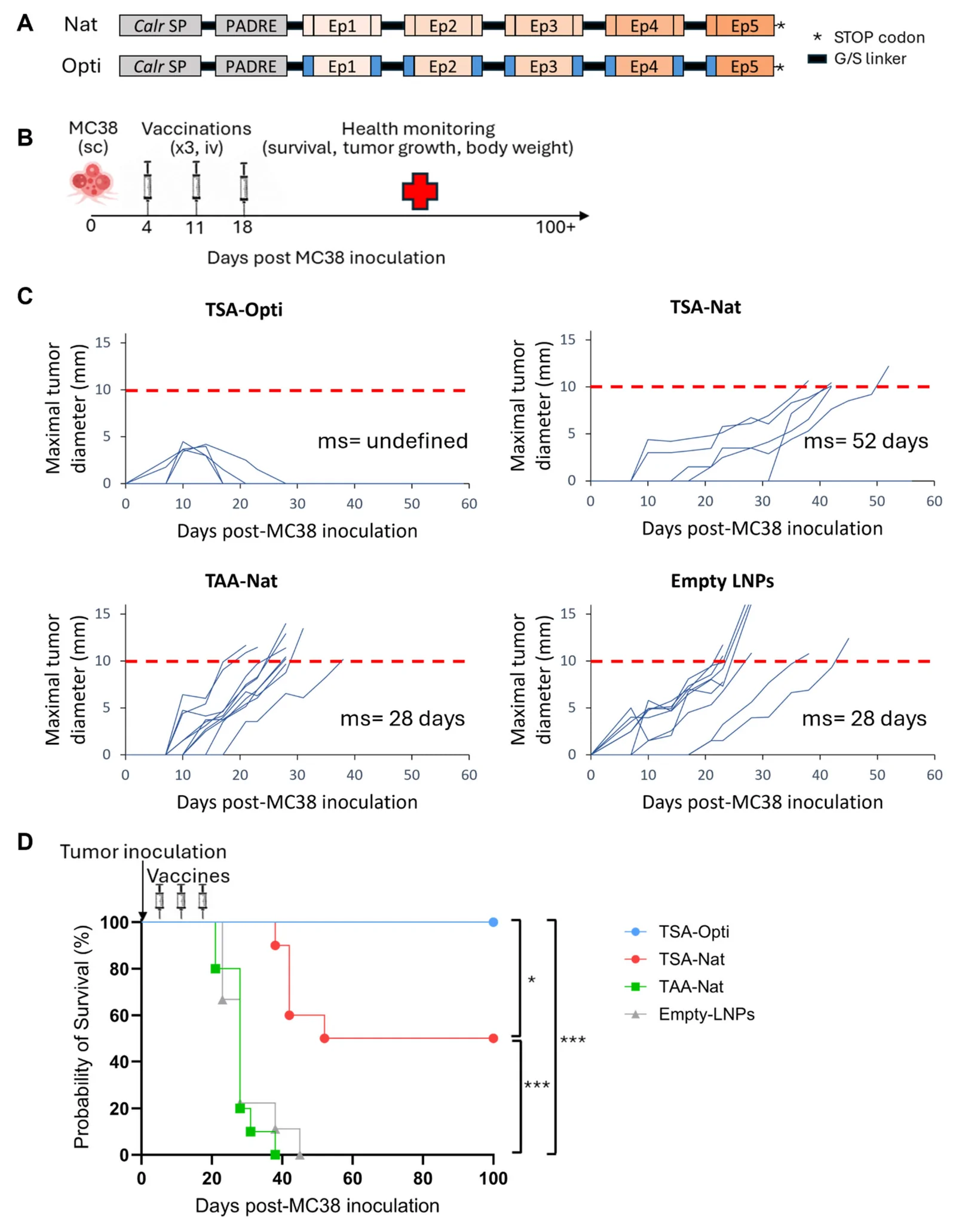
(**A**) Schematic representation of the open reading frame of the two multiepitope mRNA vaccine construct strategies: Opti, in which epitopes are flanked by the ovalbumin-derived LEQLE and TEWTS sequences (indicated in blue), and Nat, in which epitopes are flanked by their native amino acid sequences (shown in the same color as the corresponding epitope). (**B**) Experimental design of the therapeutic vaccination protocol. Mice (*n* = 10 per group) were inoculated subcutaneously with 1 × 10^5^ MC38 tumor cells on day 0 and vaccinated intravenously with LNP-formulated mRNA (TSA-Opti, TSA-Nat, TAA-Nat, or empty LNPs) on days 4, 11, and 18. Tumor growth was monitored biweekly. Humane endpoints were defined as tumor necrosis >5 mm or tumor diameter ≥10 mm (longest axis). All remaining tumor-free mice were euthanized on day 100. ms = median survival. (**C**) Tumor growth curves for individual mice in each treatment group, showing maximal tumor diameter over time. Humane endpoints were defined as tumor necrosis >5 mm or tumor diameter ≥10 mm (longest axis; red dashed lines). (**D**) Kaplan–Meier survival curves comparing mice treated with empty LNPs, TAA-Nat, TSA-Nat, or TSA-Opti vaccines. Statistical significance was assessed using a log-rank (Mantel–Cox) test (* *p* ≤ 0.05, *** *p* ≤ 0.0001).

### 3.2. Vaccination with TSA-Opti Eradicates Established Tumors in a Therapeutic Setting

The efficacy of the different vaccine constructs was first evaluated in a therapeutic setting. As illustrated in Figure 1B, vaccination started 4 days after tumor inoculation, with three weekly intravenous doses (10 μg). The mice’s health and tumor size were monitored for one hundred days. The TSA-Opti vaccine showed striking efficacy, with complete tumor control in all treated mice within 30 days (Figure 1C) and 100% survival on day 100. In contrast, the TSA-Nat vaccine delayed tumor growth but did not consistently eradicate tumors, yielding an overall survival of 50% and a median survival of 52 days, compared with 28 days for empty LNP controls. The TAA-based vaccine (Nat design) showed no benefit in tumor growth or survival, comparable to mice vaccinated with empty LNPs (Figure 1C,D).

Because all mice survived when the TSA-Opti vaccine was administered 4 days after tumor inoculation, we next sought to define the latest time point at which vaccination could still confer therapeutic benefit. To address this, the initial vaccination was initiated 4, 7, 11, or 14 days after tumor inoculation (Figure S1). Robust efficacy was maintained when vaccine initiation was delayed up to 11 days post-tumor inoculation, corresponding to approximately half of the control group’s median survival time. Even then, the TSA-Opti-vaccinated group had a median survival approximately 3-fold higher than that of the control group (68 vs. 23 days) and a 50% survival rate at the study endpoint.

These results obtained in a therapeutic setting support two important conclusions: (i) aeTSAs constitute actionable targets for the treatment of established tumors, (ii) the Opti design elicits a substantially more potent immune response than the Nat design.

### 3.3. Optimized Flanking Sequences Enhance CD8^+^ T-Cell Priming and Antigen Presentation

We next aimed to evaluate how flanking sequences influence TSA-specific CD8+ T-cell responses. Mice received three intravenous vaccine doses at one-week intervals, and spleens and lymph nodes were collected seven days after the final immunization and pooled for downstream analyses. Expansion of TSA-specific CD8^+^ T cells was measured using IFNγ ELISpots and MHC–peptide dextramers. Two aeTSAs were highly immunogenic (TSA2 and TSA4), whereas the remaining three aeTSAs did not elicit detectable immune responses under the conditions tested (Figure 2A,B). Compared with TSA-Nat, the TSA-Opti construct markedly increased the frequency of IFNγ-secreting cells, as measured by ELISPOT (Figure 2A), and enhanced splenic antigen-specific CD8^+^ T-cell expansion by 20–25-fold for both TSA2 and TSA4 (Figure 2B). Notably, TSA2- and TSA4-specific CD8+ T cells collectively accounted for ~35% of total splenic CD8+ T cells in TSA-Opti-immunized mice (Figure 2B).

To determine whether the enhanced TSA2- and TSA4-specific CD8^+^ T-cell responses induced by the TSA-Opti construct resulted from increased antigen presentation by APCs, we established an in vitro assay in which DC2.4 cells, a murine dendritic cell line, were lipofected with the TSA-encoding mRNA-LNP constructs. We focused our analyses on TSA2 and TSA4 for two reasons. First and foremost, these two TSAs were entirely responsible for the biologic activity of our vaccines (Figure 2). Second, they showed the highest MS-detectable copy numbers per cell after a 2 h pulse with synthetic peptide analogs (Figure S2). We reasoned that their superior MHC binding affinity would facilitate their detection in lipofected DC2.4 cells. We found that, relative to the TSA-Nat construct, the TSA-Opti construct induced higher abundance of MHC-I-presented epitopes per cell for both TSA2 and TSA4 (Figure 2C). Thus, at 6 h post-lipofection, TSA-Opti-transfected cells exhibited at least a 2.5-fold increase in average TSA copy number per cell relative to TSA-Nat-transfected cells. This difference was further amplified after 18 h, with TSA2 levels increasing 7.8-fold under the TSA-Opti condition. To eliminate any confounding factors, we assessed whether flanking-sequence optimization might generate alternative (non-aeTSA) MAPs. The complete TSA-Opti and TSA-Nat constructs were screened in silico with NetMHCpan 4.1b to predict 8–11-mer binders to H2-Kb and H2-Db. Predicted binders (%Rank_EL ≤ 2, *n* = 27) were compiled into a targeted LC–MS/MS inclusion list (Table S2), which was used to interrogate MHC I peptide datasets for potential alternative vaccine-derived ligands. None of these non-aeTSA MAPs were detected by targeted immunopeptidomics in DC2.4 cells following transfection with either the TSA-Opti or TSA-Nat constructs. These data indicate that OVA-derived flanking sequences increase the abundance of the intended TSA-derived peptide–MHC I complexes without producing detectable alternative vaccine-derived MHC I ligands (Table S2) within the limits of detection of the targeted MS assay.

Together, these findings position flanking-region optimization as a promising strategy to improve vaccine efficacy in vivo. In the context of TSA2 and TSA4, combining LEQLE and TEWTS flanking sequences enhances antigen processing and increases epitope presentation, resulting in stronger antigen-specific immune responses.

### 3.4. TSA-Opti Vaccination Promotes CD8^+^ T-Cell Differentiation Toward an MPEC-like Phenotype

We next used scRNA-seq to profile the transcriptional states of antigen-specific CD8^+^ T cells induced by each vaccine construct. Dextramer^+^ CD8^+^ T cells specific for TSA2 and TSA4 were isolated 7 days after the third weekly immunization. Unsupervised clustering analysis identified 10 distinct CD8^+^ T-cell clusters (Figure 3A), one of which was excluded due to low quality (see Section 2.11.2 for details). UMAPs revealed a clear separation of CD8^+^ T-cell states across conditions. In particular, cluster 3 occupied a distinct transcriptional space and emerged as significantly enriched in the Opti condition compared with the Nat condition (24% vs. 7.1%) (Figure 3B,C).

To define the identity of these clustered populations, we next examined the expression of key CD8^+^ T-cell markers across clusters (Figure 3D). Cluster 3, dominant under Opti conditions, displayed a transcriptional profile consistent with a memory precursor-like (MPEC) population, characterized by elevated expression of *Il7r*, *Cd27*, and *Tcf7*. In contrast, clusters 0–2, which were more prevalent in Nat samples, exhibited features of terminal effector cells, including strong cytotoxic signatures (*Gzma*, *Prf1*, *Zeb2*, *Klrg1*). Cluster 4, while also enriched in Nat samples and sharing overall effector-like features, showed a distinct transcriptional profile relative to clusters 0–2, including higher expression of *Itga4*, suggestive of a transitional effector state within the broader effector compartment. The remaining clusters corresponded to exhausted (cluster 5), interferon-responsive (cluster 6), quiescent/naive (cluster 7), and cycling (cluster 8) states [55].

Given the enrichment of the memory precursor-like cluster 3 under TSA-Opti, we next directly compared Opti and Nat conditions to assess broader effects on CD8^+^ T-cell differentiation. Pseudobulk differential expression analysis revealed a set of differentially expressed genes (DEGs). To further characterize these transcriptional differences, we generated a heatmap of Z-scored gene expression across the four epitope/vaccine conditions using a curated set of DEGs and canonical CD8^+^ T-cell marker genes (Figure 3E). This analysis highlighted distinct transcriptional programs, with Opti-induced CD8^+^ T cells exhibiting a tissue-trafficking-biased and memory-precursor profile (*Cxcr3, Cxcr6, Itgb1, Il7r, Tcf7*), whereas Nat conditions were associated with a lymphoid-homing and activation/stress-linked effector state (*Fos, Jun, Egr1, Cd69, Hspa1a/b, Ccr7, Gzmb, Gzmm, Klrg1*). This separation was robust but not absolute, as *Cd27* and *Mcl1* showed increased expression under TSA-Opti for TSA2 but not TSA4. Nonetheless, global transcriptomic patterns were conserved across epitopes, indicating that flanking-sequence composition exerted a dominant influence on CD8^+^ T-cell fate independently of antigen identity.

### 3.5. TSA-Opti Vaccination Increases TCR Clonal Diversity

VDJ repertoire analysis of TCRβ CDR3 sequences was performed in parallel with scRNA-seq to assess clonal diversity and evenness within TSA2- and TSA4-specific CD8^+^ T cells isolated using cognate peptide–MHC dextramers. Diversity was quantified using D50, Shannon-based (q1), and Simpson-based (q2) indices, estimated from 500 bootstrap iterations of 1000-cell subsamples to ensure robust comparisons. D50 (Figure 4A), defined as the number of clonotypes accounting for 50% of the repertoire, was higher in the Opti condition for TSA2-specific responses (11.17 vs. 6.04 in Nat), indicating reduced clonal skewing. Consistently, both q1 (Figure 4B) and q2 (Figure 4C) were increased in the TSA-Opti conditions, supporting a broader and more even CDR3β clonal distribution. Although TSA4-specific responses were less diverse overall, they also showed a modest increase in the Opti condition. Together, these data indicate that flanking sequence optimization enhances both the clonal breadth and evenness of the antigen-specific TCR repertoire. This broader recruitment of clonotypes may reflect increased peptide-MHC I density during priming, thereby allowing activation of lower-frequency or lower-avidity precursors that are less efficiently recruited by the TSA-Nat construct.

### 3.6. TSA-Opti Vaccination Promotes Intratumoral Accumulation of TSA2-Specific CD8^+^ T Cells

The strong tumor-clearing ability of the TSA-Opti vaccine prompted us to determine the abundance and specificity of the tumor-infiltrating lymphocytes (TILs). Because TSA-Opti-vaccinated mice rapidly cleared tumors (Figure 1C and Figure S1), the first vaccination was delayed to day 7 post-inoculation (instead of day 4), extending tumor presence and enabling synchronized tumor collection across groups while maintaining a three-dose regimen. Although vaccinations were delayed in this experiment, the differences in tumor sizes and weights between groups, shown in Figure 5A,B, are consistent with the vaccine performances and tumor kinetics previously described (Figure 1C). Tumor growth was rapid in naïve mice, considerably delayed in the TSA-Nat group, and only transient in the TSA-Opti group.

The interrogation of TIL specificity using dextramers revealed a clear difference in tumor infiltration between recipients of the TSA-Opti vaccines vs. the TSA-Nat vaccines (Figure 5C). Specifically, TSA2-specific cells accounted for an average of 40% of CD8+ TILs in TSA-Opti-vaccinated mice, compared with 8.6% in TSA-Nat-vaccinated counterparts. Minimal infiltration by TSA4-specific CD8+ T cells was observed in both TSA-Opti and TSA-Nat. The high abundance of infiltrating TSA2-specific CD8^+^ T cells is consistent with a major contribution of these cells to the superior tumor clearance observed in TSA-Opti-vaccinated mice. However, directly assessing their role in tumor control would require adoptive transfer of TSA2-specific T cells.

### 3.7. TSA-Opti Vaccination Induces Durable and Recallable Anti-Tumor Immunity in the Spleen and Bone Marrow

scRNA-seq revealed increased memory precursor and migration-associated programs under the TSA-Opti condition (Figure 3), accompanied by a higher number of TSA2-specific TILs (Figure 5). We therefore evaluated the durability and recall capacity of TSA-specific CD8^+^ T-cell responses in vivo.

Longitudinal dextramer staining performed on days 4, 7, 14, 30, and 60 days post-immunization (Figure 6A) revealed that TSA-Opti elicited 2- to 20-fold higher frequencies of TSA2- and TSA4-specific CD8^+^ T cells in the spleen compared to TSA-Nat (Figure 6B). These responses were more sustained and showed reduced contraction over time. These cells maintained an activated CD62L^lo^ CD44^hi^ phenotype, consistent with an effector-memory profile, indicative of enhanced persistence and higher-quality differentiation (Figure 6C).

Evidence in mice and humans suggests that the bone marrow is the most propitious site for the expansion and persistence of effector and memory T cells reactive to tumor antigens [56,57]. Notably, we observed that the bone marrow of Opti-vaccinated mice harbored a much higher proportion of TSA-specific CD8+ T cells than that of Nat-vaccinated animals, consistent with enhanced persistence. In addition, booster immunization revealed that this pool of long-lived TSA-specific CD8^+^ T cells in the bone marrow could expand upon recall, thereby supporting the establishment of a durable, recallable memory reservoir (Figure 6D).

In prophylactic settings (Figure 7A,B), TSA vaccination conferred significant protection against MC38 tumor development (100% for TSA-Opti and 90% for TSA-Nat). In contrast, tumor growth was observed in all mice receiving the TAA-Nat vaccine or empty LNPs, in line with results obtained in the therapeutic setting (Figure 1C,D). On day 90, to assess long-term immunity conferred by TSA vaccines, mice were rechallenged with 1 × 10^5^ MC38 cells. In the TSA-Nat group, five of nine tumor-free mice at rechallenge developed tumors, indicating partial loss of protection. In contrast, all TSA-Opti-vaccinated mice remained tumor-free through day 130, resulting in significantly improved survival (log-rank *p* < 0.01). Collectively, these data demonstrate that flanking-sequence optimization enhances the magnitude, durability, and recall capacity of CD8^+^ T-cell responses, resulting in superior and long-lasting antitumor immunity.

## 4. Discussion

In this study, we show that epitope-flanking sequences are active determinants of multiepitope mRNA vaccine performance. Substitution of the natural aeTSAs flanking pentamers with OVA/SIINFEKL-derived LEQLE and TEWTS increased peptide–MHC I presentation in mRNA-lipofected APCs and enhanced the expansion, functionality, clonal diversity, tissue trafficking, persistence, and recall capacity of antigen-specific CD8^+^ T cells. These effects were observed for the two epitopes that elicited detectable antigen-specific responses under the conditions tested, with a greater magnitude for TSA2 than for TSA4. The flanking sequences used in the vaccine could thereby make the difference between life and death for mice bearing MC38 tumors.

The enhanced presentation observed with the Opti sequences may reflect more favorable sequence-dependent processing and trimming properties of the flanking regions. Because the constructs contain an N-terminal calreticulin signal peptide, this effect could potentially arise downstream of ER targeting through differences in proteolytic processing and peptide trimming, including ER aminopeptidase activity. Indeed, recent work using the SIINFEKL model has demonstrated that exceptionally efficient ERAP1-dependent processing of an epitope can substantially influence its abundance within the MHC-I immunopeptidome [30].

The in vivo experiments provide a central functional validation of these findings. In a therapeutic setting, when immunization was initiated early after tumor implantation, TSA-Opti vaccination improved survival compared with TSA-Nat. Importantly, the therapeutic efficacy was maintained when vaccination was initiated at a later stage of tumor progression (day 11 post-implantation), despite the rapid growth kinetics of the MC38 model (median survival ~21 days) (Figure S1). In addition, prophylactic vaccination with TSA-Opti conferred complete long-term protection, with complete survival upon tumor rechallenge on day 90. Only 50% of mice vaccinated with TSA-Nat survived after rechallenge. These results demonstrate that epitope-sequence context directly modulates vaccine efficacy and that LEQLE and TEWTS flanking sequences enhance both the potency and durability of antitumor immunity.

To further contextualize these findings, we compared responses elicited by aeTSAs and TAAs. All vaccine-encoded MHC I antigens were initially identified by mass spectrometry on MC38 tumor cells, confirming their endogenous presentation. Despite this, the vaccine encoding TAAs with natural flanking sequences conferred no survival benefit, whereas aeTSA-based vaccines were effective. This is consistent with the limited immunogenicity of many TAAs, which are subject to central tolerance [5]. This negative control group is highly relevant in light of recent studies, in humans and mice, showing that vaccination with RNA-LNPs that do not encode tumor antigens can enhance antitumor responses via type I interferon-dependent mechanisms that trigger a cascade leading to epitope spreading [58,59,60,61]. In our model, vaccine efficacy was determined by two factors: the nature of antigens (TAAs vs. aeTSAs) and the type of flanking sequences (TSA-Opti vs. TSA-Nat). Thus, the anti-tumor effect in our model was not a bystander effect but rather resulted from adequate presentation of immunogenic aeTSAs.

Quantitative immunopeptidomics demonstrated increased TSA-derived MAPs in APCs lipofected with TSA-Opti compared with TSA-Nat constructs, providing direct evidence that flanking-sequence optimization enhances antigen presentation. These changes in antigen presentation were associated with coordinated shifts in T-cell state. TSA-Opti was associated with a memory- and trafficking-associated program, characterized by increased expression of Il7r, Cxcr3, and Cxcr6, together with reduced expression of effector- and activation-associated genes such as Klrg1 and Gzmb, without compromising antitumor efficacy in vivo. In contrast, TSA-Nat induced a more activated effector profile. These findings are consistent with models in which antigen density and duration of presentation shape CD8^+^ T-cell differentiation [62,63,64]. However, the present study does not establish that the MPEC-like transcriptional program directly contributes to tumor control.

A practical implication of these findings is that quantitative immunopeptidomics can be used as a preclinical screening platform for multiepitope mRNA vaccine design. Rather than selecting flanking sequences solely from endogenous source proteins or relying solely on in silico prediction, candidate constructs can be transfected into APCs and ranked directly by the abundance of intended peptide–MHC I complexes. This approach could identify epitope–flank combinations that maximize presentation while excluding constructs that generate unintended vaccine-derived ligands. Such a workflow would provide an experimentally grounded strategy to prioritize vaccine architectures before in vivo testing.

Epitope-flanking sequences also influenced T-cell localization, with increased tumor infiltration and bone marrow persistence associated with TSA-Opti, along with upregulation of tissue-trafficking genes, including Cxcr3, Cxcr6, and Itgb1. In contrast, TSA-Nat was associated with a lymphoid-homing profile marked by Ccr7. In addition, TSA-Opti increased repertoire evenness, indicating reduced clonal dominance and broader recruitment of antigen-specific precursors. Although the antigen specificity of individual TCR clonotypes was not experimentally validated, this may reflect enhanced antigen presentation, enabling priming of a wider range of T-cell clones and thereby increasing response diversity and robustness [65].

Despite these advances, further work is needed to fully elucidate the mechanisms by which flanking sequences modulate antigen processing and presentation. While our data demonstrate increased peptide–MHC abundance with the TSA-Opti construct, the relative contribution of peptide trimming and MHC loading efficiency remains to be defined. In addition, only two epitopes were evaluated, limiting the generalizability of these findings. Importantly, all experiments were performed in murine models, and whether the effects of epitope-flanking sequences observed here translate to human antigen processing and vaccine efficacy remains to be established. The mRNA-LNP vaccines were administered intravenously rather than intramuscularly, as used for currently approved mRNA-LNP vaccines. Intravenous administration was selected because the LNP formulation used in this study is designed for splenic targeting, a strategy that can efficiently promote antigen presentation by professional antigen-presenting cells and robust CD8^+^ T-cell responses [51]. Whether the effects of flanking-sequence optimization are maintained following intramuscular administration remains to be determined. Future studies should evaluate additional flanking sequences and extend this approach across a broader range of epitopes and administration routes to assess its robustness and large-scale applicability. Further work may show that the best flanking sequences and intracellular antigen routing strategies differ across epitopes. Whether sequence-context optimization could similarly influence antigen processing in peptide-based vaccines, including long synthetic peptides, remains an interesting question for future investigation. Nonetheless, our study has two main implications. First, it argues that using natural flanking sequences in multiepitope vaccines is a suboptimal strategy and that several flanking sequences, such as LEQLE and TEWTS, should be tested. Second, it suggests that quantitative mass spectrometry analyses of dendritic cells lipofected with RNA-LNPs are a powerful tool for identifying optimal flanking sequences and predicting in vivo immunogenicity.

## 5. Conclusions

This study identifies epitope-flanking sequences as actionable design elements in multiepitope mRNA vaccines. By increasing MAP density, OVA-derived flanks enhanced CD8^+^ T-cell expansion, clonal breadth, tissue trafficking, memory persistence, and antitumor protection. These findings support a vaccine-engineering strategy in which quantitative immunopeptidomics is used to select epitope–flank combinations that maximize presentation of intended tumor antigens while minimizing unintended vaccine-derived ligands.

## 6. Patents

G.O.-L., M.-P.H., P.T., and C.P. are named inventors on a US provisional patent application filed by the Université de Montréal and Epitopea covering flanking sequences reported in this article (patent application No. 64/098,772, titled “Vaccine constructs for the expression of MHC antigens”).

## Figures and Tables

**Figure 2:**
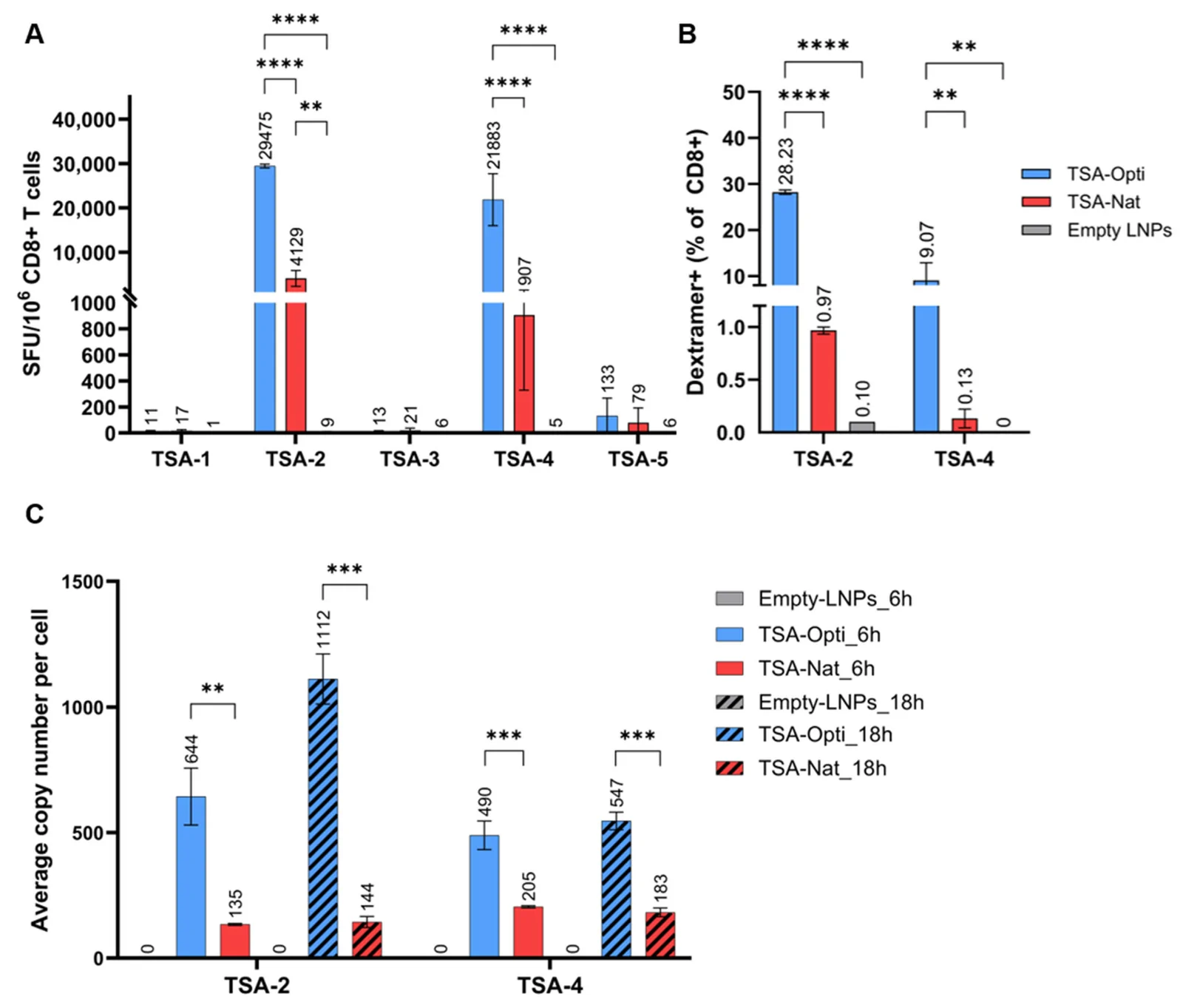
Optimized flanking sequences enhance CD8^+^ T-cell priming and antigen presentation. (**A**) IFNγ ELISpot analysis of antigen-specific CD8^+^ T-cell responses measured 7 days after the third vaccination in mice immunized with TSA-Opti or TSA-Nat vaccines. (**B**) Frequency of TSA-specific CD8^+^ T cells detected by MHC I dextramer staining on day 7 post third vaccination. No dextramer-positive cells were detected for TSA-1, TSA-3, and TSA-5. (**A**,**B**) The data shown represent three independent experiments, each consisting of three pooled mice per group. Statistical significance was assessed by two-way ANOVA with Tukey’s multiple comparisons test (**** *p* < 0.0001, ** *p* < 0.01). Error bars represent the standard deviation between the three experiments. (**C**) Quantification of TSA2 and TSA4 presentation by MHC I in DC2.4 cells following in vitro lipofection with TSA-Opti or TSA-Nat mRNA vaccines, measured by targeted mass spectrometry at the indicated time points. Three independent biological replicates were analyzed for each condition. Error bars represent the standard deviation between biological replicates. Statistical significance was assessed using Student’s *t*-test assuming equal variance and bilateral distribution (** *p* ≤ 0.01, *** *p* ≤ 0.001).

**Figure 3:**
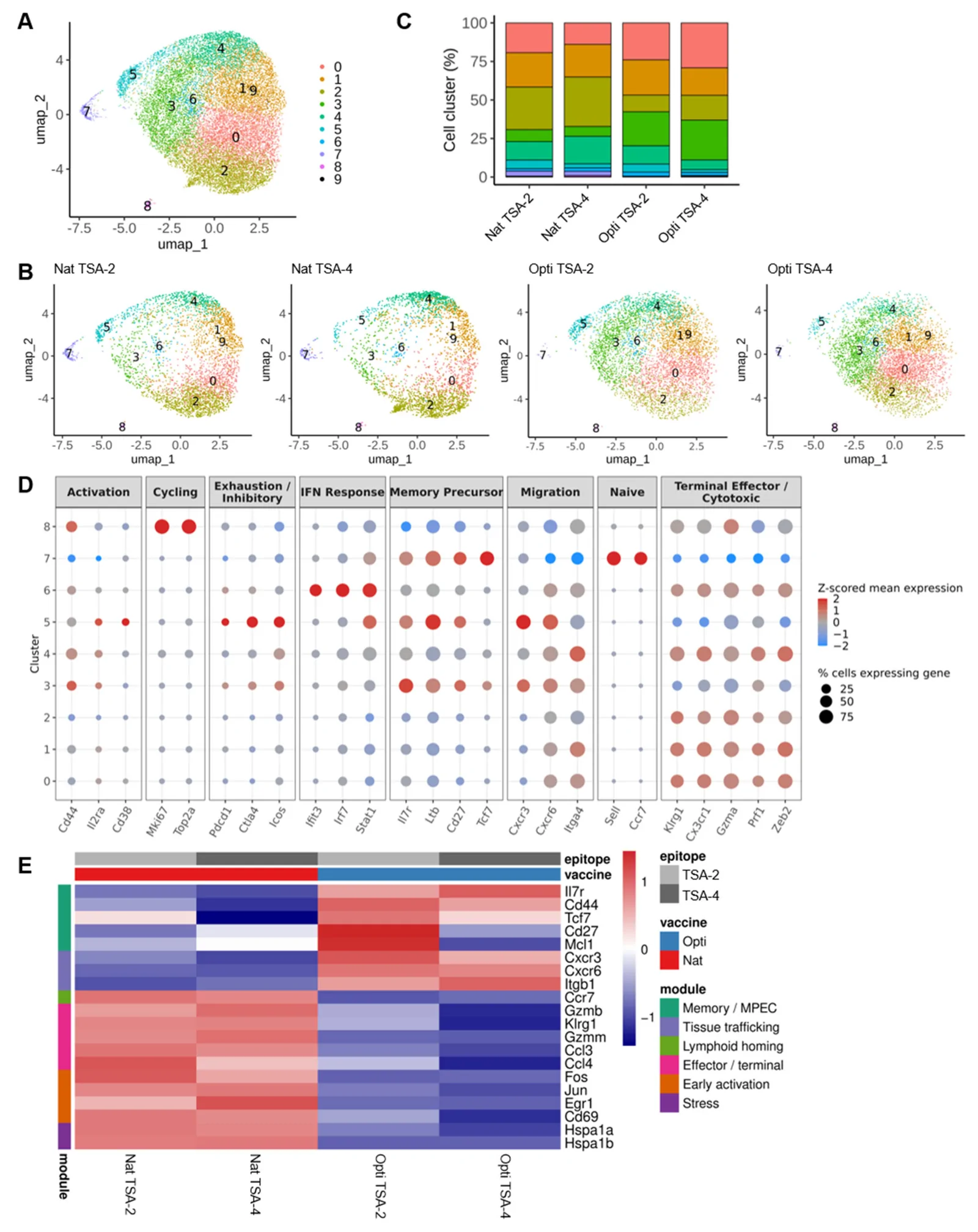
TSA-Opti vaccination biases CD8^+^ T-cell fate. (**A**,**B**) UMAPs of single-cell RNA-seq data from dextramer^+^ CD8^+^ T cells specific for TSA2 or TSA4 following vaccination with TSA-Opti or TSA-Nat (Opti TSA2, Opti TSA4, Nat TSA2, Nat TSA4), shown by transcriptional clusters. (**C**) Stacked histogram showing the distribution of clusters across samples. Panels B and C share the same color legend as panel A.(**D**) Z-scored expression of CD8^+^ T-cell gene signatures across clusters, displayed as a balloon plot. Each column represents a gene grouped by functional category, and each row corresponds to a cluster. Dot size indicates the proportion of cells within each cluster expressing the gene, while color represents the z-scored mean expression. (**E**) Heatmap of mean gene expression across conditions, Z-scored per gene (row-wise). Expression values were derived from log-normalized scRNA-seq data and averaged per condition (Opti TSA-2, Opti TSA-4, Nat TSA-2, Nat TSA-4). Genes are grouped into functional modules.

**Figure 4:**
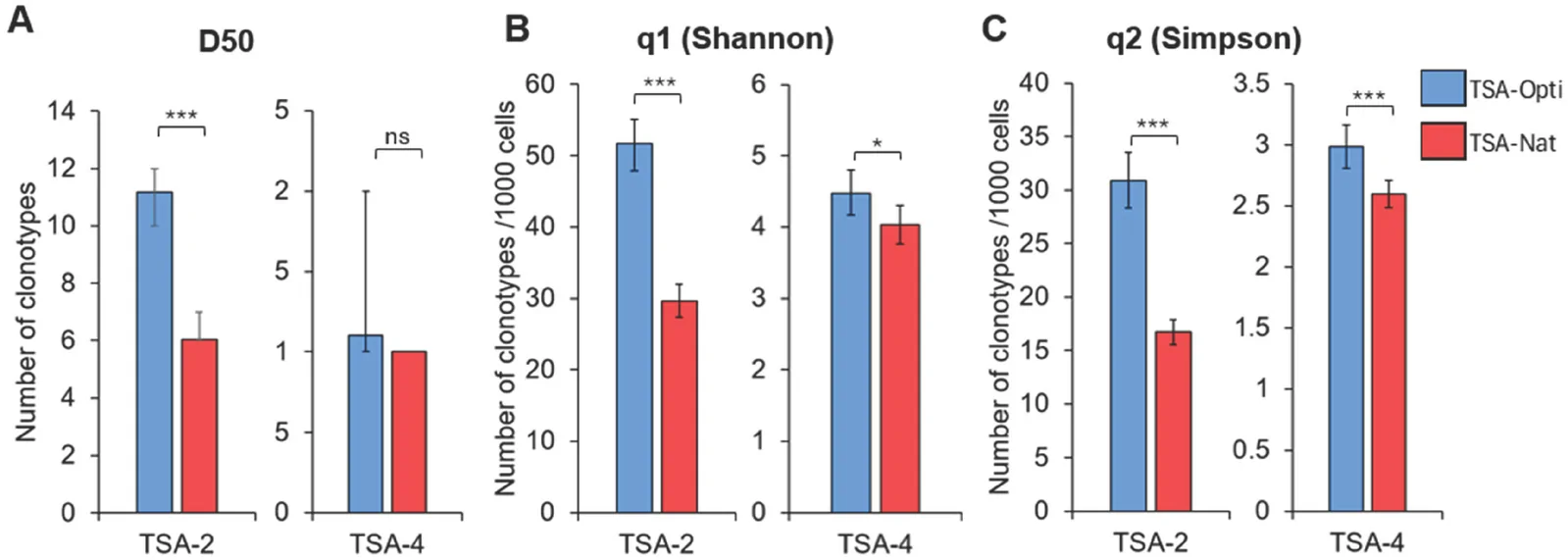
(**A**–**C**) Comparison of CDR3β repertoire diversity and evenness between TSA-Opti and TSA-Nat vaccine-induced CD8^+^ T cells. Each plot compares the TSA-Opti and TSA-Nat vaccine conditions. Bars represent the mean of the indicated diversity metric, and error bars show the 95% confidence interval calculated from 500 bootstrap iterations, each using 1000 cells as subsamples. Pairwise comparisons between vaccines were performed by calculating the proportion of bootstrap replicates in which one condition exceeded the other, yielding a two-sided empirical *p*-value. (**A**) D50: Number of clonotypes representing 50% of total cells in the rarefied sample. (**B**) q1 (exponential Shannon index): Effective number of clonotypes accounting for diversity weighted by abundance. (**C**) q2 (inverse Simpson index): Effective number of dominant clonotypes (* *p* ≤ 0.05, *** *p* ≤ 0.001, ns: not significant).

**Figure 5:**
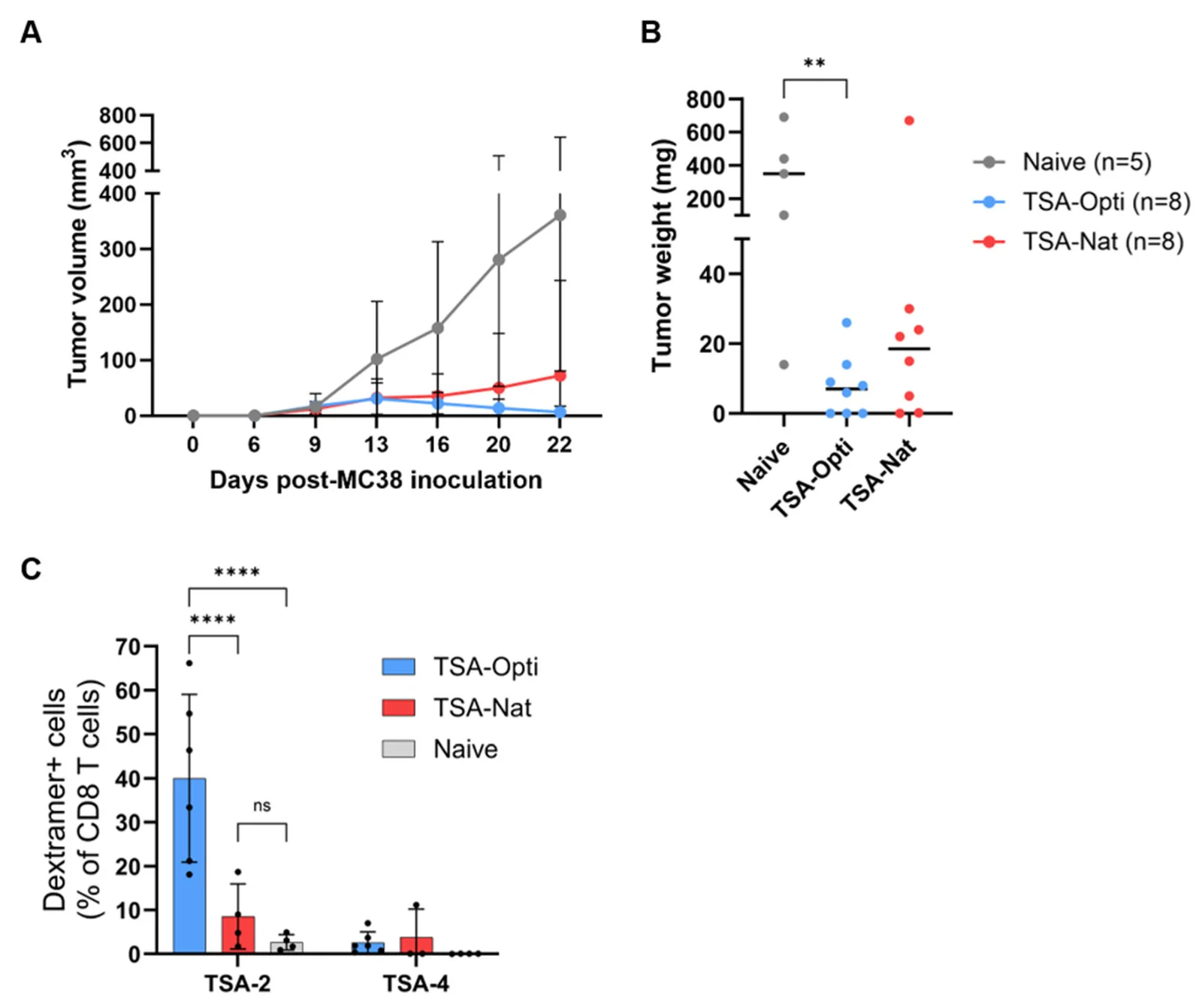
TSA-Opti vaccination promotes intratumoral accumulation of TSA2-specific CD8^+^ T cells. (**A**) Tumor growth curves following MC38 inoculation on day 0 and therapeutic vaccinations on days 7, 14, and 21 with TSA-Opti or TSA-Nat. (**B**) Tumor weights were measured at the time of harvest. Statistical significance was assessed by an unpaired *t*-test, ** *p* < 0.01. (**C**) Frequency of TSA2-specific CD8^+^ tumor-infiltrating lymphocytes (TILs), quantified by dextramer staining. Statistical significance was assessed by two-way ANOVA with Tukey’s multiple comparisons test (**** *p* < 0.0001, ns=not significant).

**Figure 6:**
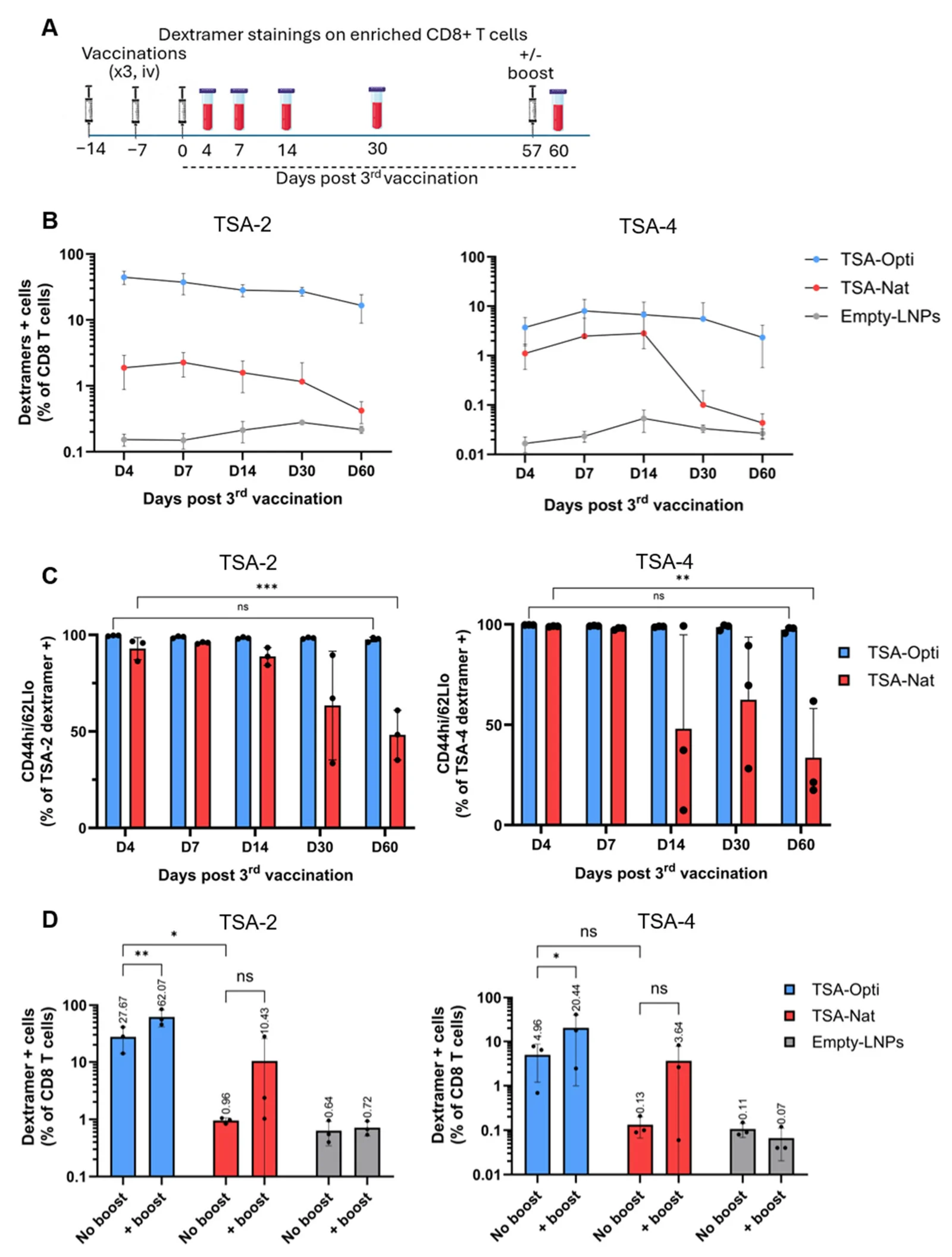
TSA-Opti vaccination induces durable and recallable anti-tumor immunity. (**A**) Schematic of the experimental design used to assess immune response kinetics and booster-induced recall responses in vaccinated mice. (**B**) Longitudinal frequencies of TSA2-specific (**left**) and TSA4-specific (**right**) CD8^+^ T cells in the spleen following vaccination with TSA-Opti or TSA-Nat, assessed by dextramer staining. (**C**) Activation and differentiation status of TSA2-specific (**left**) and TSA4-specific (**right**) CD8^+^ T cells over time, assessed by CD44 and CD62L expression. (**D**) Detection of long-lived TSA2-specific (**left**) and TSA4-specific (**right**) CD8^+^ T cells in the bone marrow following booster immunization, assessed by dextramer staining. Statistical significance was assessed using two-way ANOVA with Tukey’s multiple comparisons test (* *p* < 0.05, ** *p* < 0.01, *** *p* < 0.001, ns= not significant) (**C**,**D**).

**Figure 7:**
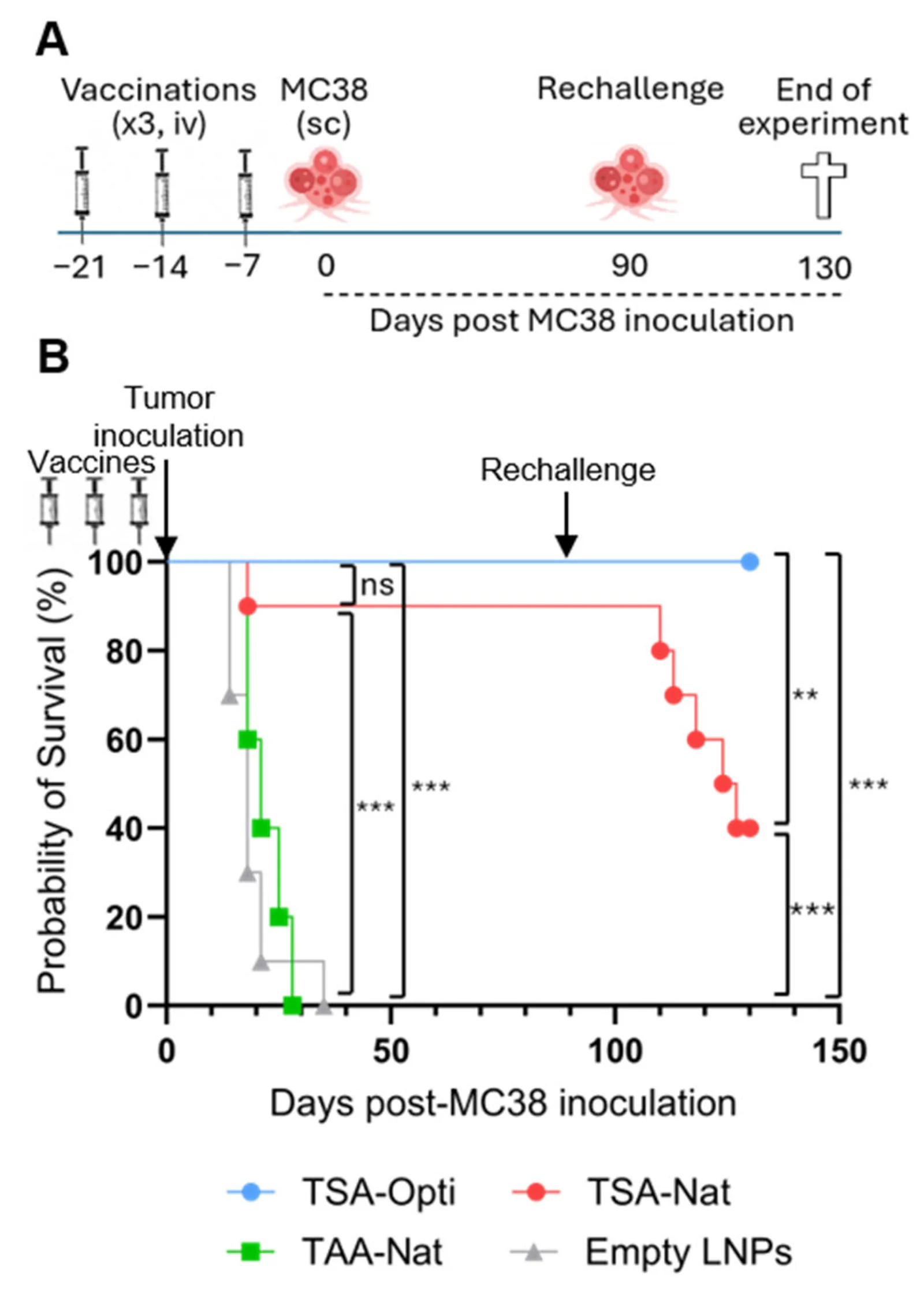
TSA-Opti vaccination induces long-term protection. (**A**) Experimental design of the prophylactic vaccination protocol. Mice (*n* = 10 per group) were vaccinated intravenously with mRNA-LNPs (TSA-Opti, TSA-Nat, TAA-Nat, or empty LNPs) on days −21, −14, and −7 and inoculated subcutaneously with 1 × 10^5^ MC38 tumor cells on day 0 and day 90 (rechallenge). Tumor growth was monitored biweekly. Humane endpoints were defined as tumor necrosis >5 mm or tumor diameter ≥10 mm (longest axis). All remaining tumor-free mice were euthanized on day 130. (**B**) Kaplan–Meier survival curves comparing mice treated prophylactically with empty LNPs, TAA-Nat, TSA-Nat, or TSA-Opti vaccines and showing long-term protection following tumor rechallenge in TSA-Opti-vaccinated mice vs. naive mice. Statistical significance was assessed using a log-rank (Mantel–Cox) test (** *p* ≤ 0.01, *** *p* ≤ 0.001, ns= not significant).

**Table 1:** Tumor antigens identified in the MC38 cell line.

Peptide Sequence	TA Type	Peptide ID	MHC Allele	NetMHCpan-4.1b rank_EL	Position	Strand	Biotype	Gene Name	Overlap with ERE
ATQQFQQL	aeTSA	TSA1	H-2-Kb	0.073	chr8:123426844-123426867	-	Intergenic	NA	MuLV-int
KSPWFTTL	aeTSA	TSA2	H-2-Kb	0.013	chr8:123426538-123426561	-	Intergenic	NA	MuLV-int
NVQEYNHL	aeTSA	TSA3	H-2-Kb	0.555	chr16:48514665-48514688	+	Exon–In frame	Morc1	
NSLRYISL	aeTSA	TSA4	H-2-Kb	0.050	chr10:109695634-109695657	-	Intron	Nav3	
SAVKNGELH	aeTSA	TSA5	H-2-Db	0.609	chrX:30833622-30833648	-	Intron	Gm10487	
SAPKGYTTF	TAA	TAA1	H-2-Db	0.028	chr4:140769464-140769490	-	Exon–In frame	Padi4	
SQFSNLQYL	TAA	TAA2	H-2-Db	0.002	chr4:144394634-144394660	-	Exon–In frame	Pramel13	
TSPLGAEKI	TAA	TAA3	H-2-Db	0.194	chr12:104961569-104961595	-	Exon–In frame	Syne3	
IAIGNPVHL	TAA	TAA4	H-2-Db	0.002	chr3:5245523-5245549	+	Exon–In frame	Zfhx4	
RGPSNAFFL	TAA	TAA5	H-2-Db	0.008	chr6:128242413-128242439	-	Exon–In frame	Tead4	

## Data Availability

The MC38 RNA-seq data generated and analyzed in the present study have been deposited in GEO and are accessible under accession number GSE335626. The MC38 MS data have been deposited to the ProteomeXchange Consortium via the PRIDE partner repository with the dataset identifier PXD079436.

## Supplementary Materials

The following supporting information can be downloaded at: https://www.mdpi.com/article/10.3390/cells15191753/s1, Table S1. Nucleotide sequence of vaccine constructs. Table S2. Predicted H-2K^b^/H-2D^b^-binding 8–11mer peptides (NetMHCpan4.1b, rank ≤ 2%) from TSA-Opti and TSA-Nat constructs used to assess generation of vaccine-derived alternative sequences by targeted MS. Figure S1. Effect of delayed therapeutic vaccination on TSA-Opti–mediated tumor control. Figure S2. Quantification of aeTSA cell surface abundance following peptide pulsing. Figure S3. Differential gene expression in TSA-specific CD8^+^ T cells following TSA-Opti versus TSA-Nat vaccination.

## Abbreviations

The following abbreviations are used in this manuscript:

MAP	MHC I-associated peptide
TA	Tumor antigen
TSA	Tumor-specific antigen
aeTSA	Aberrantly expressed tumor-specific antigen
mTSA	Mutated tumor-specific antigen
TAA	Tumor-associated antigen
APC	Antigen-presenting cell
DC	Dendritic cell
OVA	Ovalbumin
LNP	Lipid nanoparticle
TRB	T-cell receptor (TCR) beta chain
