# Supplementary material for: Optimized Epitope-Flanking Sequences Enhance Antigen Presentation and T-Cell Responses Following mRNA Vaccination
Source: Cells. 2026 Sep 26;15(19):1753. doi: 10.3390/cells15191753 (PMC13664092; doi:10.3390/cells15191753)
Supplement: Supplementary file 1 [file cells-15-01753-s001.zip › cells-4527361-supplementary.pdf]

Supplementary figures and tables

Table S1. Nucleotide sequences of the vaccine construct ORFs

Vaccine	Nucleotide ORF sequence
TSA-Nat	AUGCUGCUGAGCGUGCCACUGCUGCUGGGCCUGCUGGGACUGGCCGUGGCCGGC<br>GGAGGCGGCUCCGGUGGCGGGGGCUCUGCCAAGUUUGUGGCCGCCUGGACCCUG<br>AAGGCUGCUGCCGGAGGCGGAGGAUCUGGCGGUGGCGGCAGCACCACCGCCCUG<br>GUGGCCACCCAGCAGUUUCAGCAGCUGCAGGCAGCCAUGCACGGGGGCGGAGGC<br>UCUGGAGGAGGAGGCUCCGAGGGCCUCUUCAACAAGUCUCCAUGGUUUACCACC<br>CUCAUCUCCACCAUCAUGGGUGGCGGGGGCAGUGGCGGAGGCGGCAGCAAGCAG<br>GAGUUUCUGAACGUGCAGGAGUACAACCAUCUCCUGAAGGUGAUGGGCGGAGG<br>AGGUGGCUCUGGCGGGGGCGGCUCCUCCAGGGAUGAGGUGAACUCCCUGCGCUA<br>CAUCUCCCUGCAGGGAGGCGGAGGAUCUGGAGGUGGCGGCAGCACCAGCGCUGU<br>GAAGAACGGAGAGCUGCACUAA
TSA-Opti	AUGCUGCUGAGCGUGCCACUGCUGCUGGGCCUGCUGGGACUGGCCGUGGCCGGC<br>GGAGGCGGCUCCGGUGGCGGGGGCUCUGCCAAGUUUGUGGCCGCCUGGACCCUG<br>AAGGCUGCUGCCGGAGGCGGAGGAUCUGGCGGUGGCGGCAGCCUGGAGCAGCUG<br>GAGGCCACCCAGCAGUUUCAGCAGCUGACCGAGUGGACCAGCGGGGGCGGAGGC<br>UCUGGAGGAGGAGGCUCCCUGGAGCAGCUGGAGAAGUCUCCAUGGUUUACCACC<br>CUCACCGAGUGGACCAGCGGUGGCGGGGGCAGUGGCGGAGGCGGCAGCCUGGAG<br>CAGCUGGAGAACGUGCAGGAGUACAACCACCUGACCGAGUGGACCAGCGGAGGA<br>GGUGGCUCUGGCGGGGGCGGCUCCCUGGAGCAGCUGGAGAACUCCCUGCGCUAC<br>AUCUCCCUCACCGAGUGGACCAGCGGAGGCGGAGGAUCUGGAGGUGGCGGCAGC<br>CUGGAGCAGCUGGAGAGCGCUGUGAAGAACGGAGAGCUGCACUAA

Table S2. Predicted H-2K^b/H-2D^b-binding 8-11mer peptides (NetMHCpan4.1b, rank ≤2%) from TSA-Opti and TSA-Nat constructs used to assess generation of vaccine-derived alternative sequences by targeted MS.

Peptide	MHC Allele	%Rank_EL Kb	%Rank_EL Db	Bind Level Kb	Bind Level Db	Vaccine(s)	Origin in construct
LSVPLLLGL	H-2-Kb	0.951	NA	WB	NA	Opti/Nat	Signal peptide
SVPLLLGLL	H-2-Kb, H-2-Db	0.577	1.284	WB	WB	Opti/Nat	Signal peptide
EATQQFQQL	H-2-Kb	1.776	NA	WB	NA	Opti	flank+TSA1
EKSPWFTTL	H-2-Kb	1.856	NA	WB	NA	Opti	flank+TSA2
KSPWFTTLT	H-2-Kb	1.445	NA	WB	NA	Opti	TSA2+flank
ENVQEYNHL	H-2-Kb	1.504	NA	WB	NA	Opti	flank+TSA3
ENSLRYISL	H-2-Kb	1.074	NA	WB	NA	Opti	flank+TSA4
SVPLLLGL	H-2-Kb	0.293	NA	SB	NA	Opti/Nat	Signal peptide
FNKSPWFTTL	H-2-Kb	1.852	NA	WB	NA	Nat	flank+TSA2
TALVATQQF	H-2-Kb, H-2-Db	1.943	0.83	WB	WB	Nat	flank+TSA1
VATQQFQQL	H-2-Kb, H-2-Db	0.141	0.869	SB	WB	Nat	flank+TSA1
NKSPWFTTL	H-2-Kb	1.444	NA	WB	NA	Nat	flank+TSA2
KSPWFTTLI	H-2-Kb, H-2-Db	0.264	0.647	SB	WB	Nat	TSA2+flank
LNVQEYNHL	H-2-Kb	0.357	NA	SB	NA	Nat	flank+TSA3
NVQEYNHLL	H-2-Kb	1.698	NA	WB	NA	Nat	TSA3+flank
SSRDEVNSL	H-2-Kb	1.079	NA	WB	NA	Nat	linker+flank+TSA4
VNSLRYISL	H-2-Kb	0.072	NA	SB	NA	Nat	flank+TSA4
SKQEFLNV	H-2-Kb	1.123	NA	WB	NA	Nat	linker+flank+TSA3
YNHLLKVM	H-2-Kb	1.933	NA	WB	NA	Nat	TSA3+flank
SLEQLENVQEY	H-2-Db	NA	0.888	NA	WB	Opti	linker+flank+TSA3
SAKFVAAWTL	H-2-Db	NA	1.886	NA	WB	Opti/Nat	linker+PADRE
EQLENSLRYI	H-2-Db	NA	0.297	NA	SB	Opti	flank+TSA4
QQFQQLTEW	H-2-Db	NA	1.856	NA	WB	Opti	TSA1+flank
GSLEQLENV	H-2-Db	NA	1.877	NA	WB	Opti	linker+flank+TSA3
EQLENVQEY	H-2-Db	NA	1.098	NA	WB	Opti	flank+TSA3
SAVKNGEL	H-2-Db	NA	0.119	NA	SB	Opti/Nat	TSA5
VQEYNHLLKVM	H-2-Db	NA	1.59	NA	WB	Nat	TSA3+flank

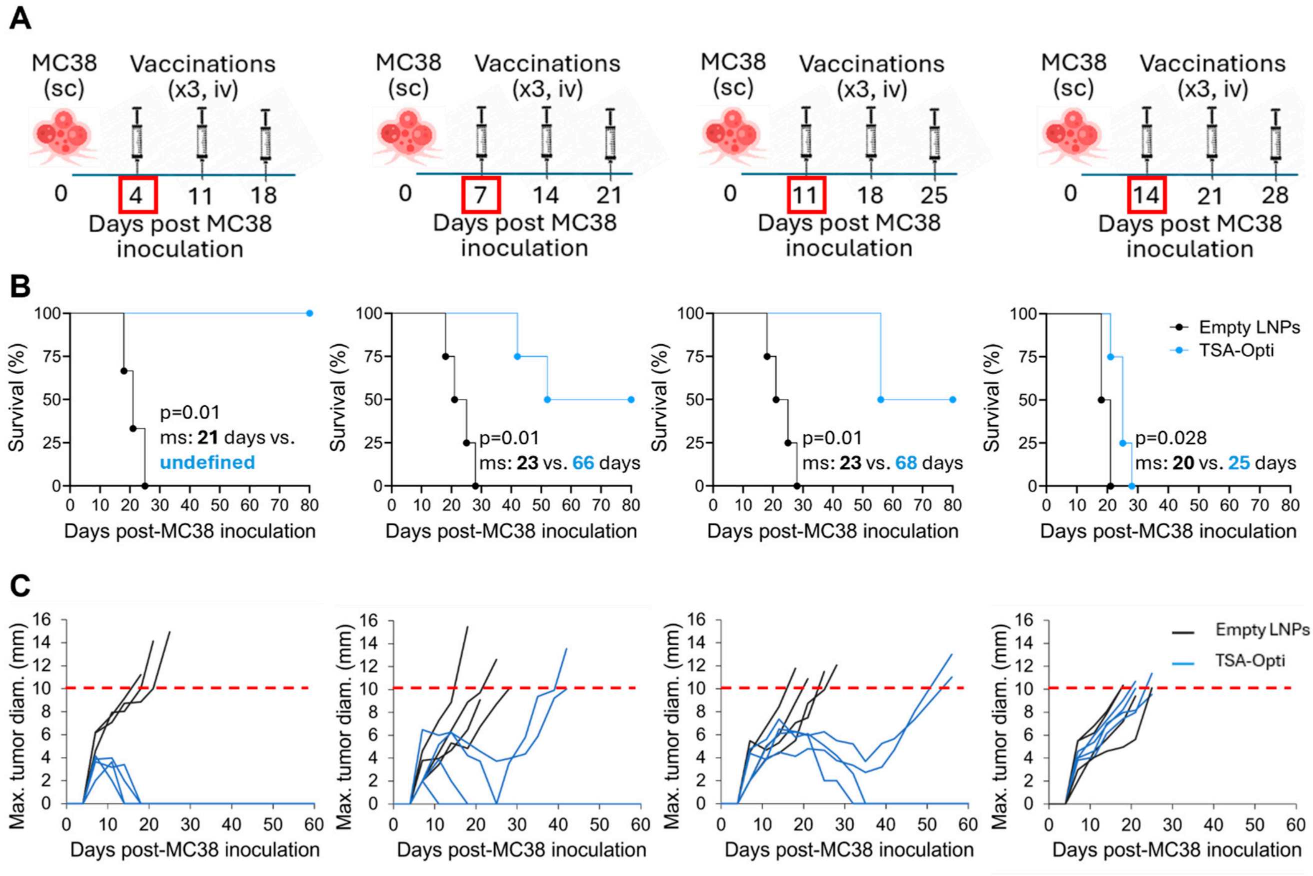

Figure S1. Effect of delayed therapeutic vaccination on TSA-Opti–mediated tumor control. (**A**) Experimental design of the delayed therapeutic vaccination protocol. Mice were inoculated subcutaneously with $1x10^5$ MC38 tumor cells at day 0 and vaccinated intravenously with LNP-formulated mRNA (TSA-Opti or empty LNPs). Mice were vaccinated three times, one week apart, starting on day 4, day 7, day 11, or day 14 post-tumor inoculation (see red boxes). (**B**) Kaplan–Meier survival curves comparing mice treated with empty LNPs or TSA-Opti vaccines. (**C**) Tumor growth curves for individual mice in each treatment group, showing maximal tumor diameter over time. Tumor growth was monitored biweekly. Humane endpoints were defined as tumor necrosis >5 mm or tumor diameter ≥10 mm (longest axis, red dashed lines). All remaining tumor-free mice were euthanized on day 100.

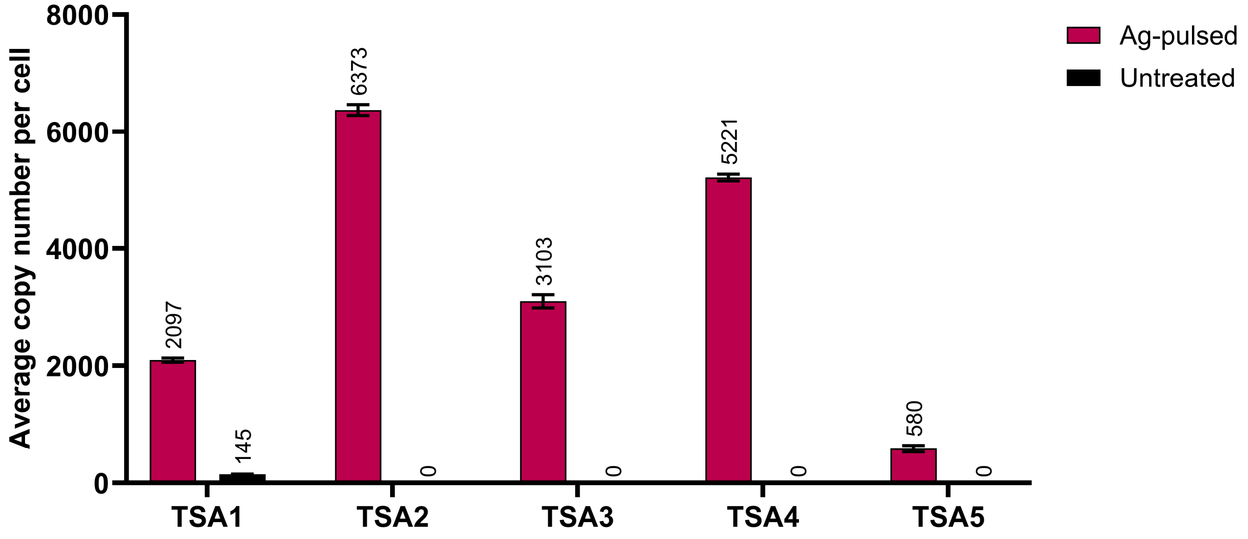

Figure S2. Quantification of aeTSA cell surface abundance following peptide pulsing. Bar plot showing cell surface abundance of each aeTSA (copy number per cell) after 2 h pulsing of DC2.4 cells with individual synthetic peptide analogs (2 µg/mL) in a non-competitive setting (each peptide was pulsed separately, and equal numbers of cells were pooled prior to MS injection). Abundance was quantified by targeted mass spectrometry. Abundance was quantified by targeted mass spectrometry.

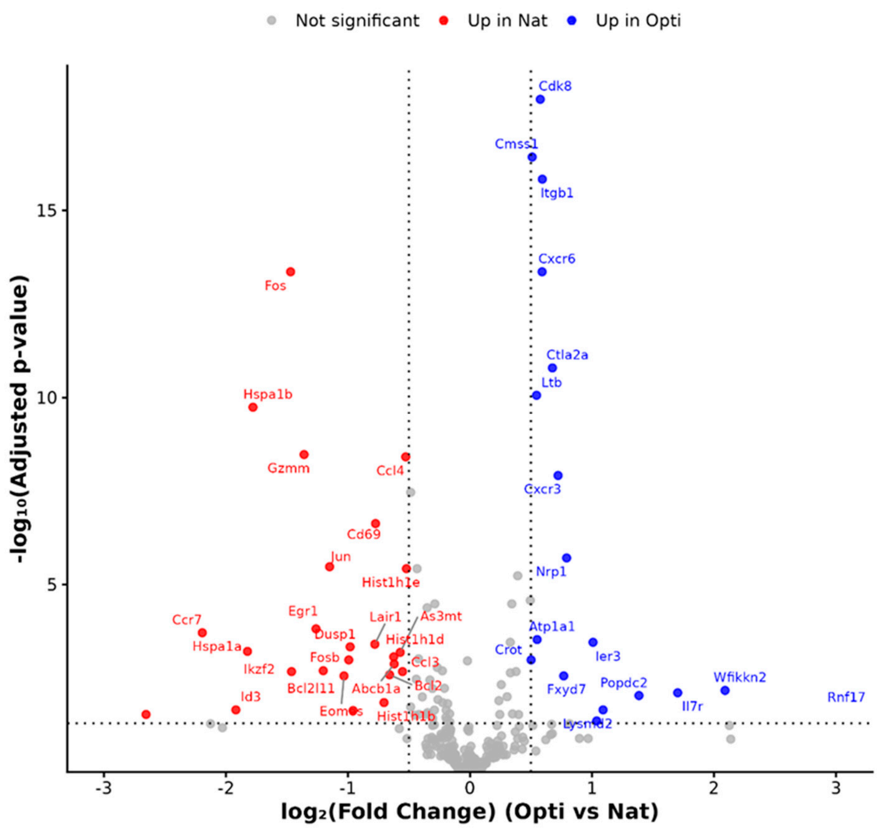

Figure S3. Differential gene expression in TSA-specific CD8$^+$ T cells following TSA-Opti versus TSA-Nat vaccination. Volcano plot showing genes differentially expressed ($\log_2$FC > 0.5, $p_{adj} < 0.05$) in TSA-specific CD8$^+$ T cells following vaccination with TSA-Opti (blue dots, 18 genes upregulated) or TSA-Nat (red dots, 25 genes upregulated).
